# A simple and sensitive detection of the binding ligands by using the receptor aggregation and NMR spectroscopy: a test case of the maltose binding protein

**DOI:** 10.1007/s10858-021-00381-x

**Published:** 2021-09-15

**Authors:** Young Kee Chae, Yoonjin Um, Hakbeom Kim

**Affiliations:** Department of Chemistry, Sejong University, 209 Neungdong-Ro, Gwangjin-Gu, Seoul, 05006 Korea

**Keywords:** Ligand screening, Protein aggregates, NMR

## Abstract

Protein-ligand interaction is one of the highlights of molecular recognition. The most popular application of this type of interaction is drug development which requires a high throughput screening of a ligand that binds to the target protein. Our goal was to find a binding ligand with a simple detection, and once this type of ligand was found, other methods could then be used to measure the detailed kinetic or thermodynamic parameters. We started with the idea that the ligand NMR signal would disappear if it was bound to the non-tumbling mass. In order to create the non-tumbling mass, we tried the aggregates of a target protein, which was fused to the elastin-like polypeptide. We chose the maltose binding proteinas a test case, and we tried it with several sugars, which included maltose, glucose, sucrose, lactose, galactose, maltotriose, and β-cyclodextrin. The maltose signal in the H-1 NMR spectrum disappeared completely as hoped around the protein to ligand ratio of 1:3 at 298 K where the proteins aggregated. The protein signals also disappeared upon aggregation except for the fast-moving part, which resulted in a cleaner background than the monomeric form. Since we only needed to look for a disappearing signal amongst those from the mixture, it should be useful in high throughput screening. Other types of sugars except for the maltotriose and β-cyclodextrin, which are siblings of the maltose, did not seem to bind at all. We believe that our system would be especially more effective when dealing with a smaller target protein, so both the protein and the bound ligand would lose their signals only when the aggregates formed. We hope that our proposed method would contribute to accelerating the development of the potent drug candidates by simultaneously identifying several binders directly from a mixture.

**Supplementary Information:**

The online version contains supplementary material available at 10.1007/s10858-021-00381-x.

## Introduction

Proteins can recognize other proteins or small molecules by utilizing specific attractive forces, such as hydrogen bonding, van der Waals contact, or hydrophobic interactions (Bissantz et al. 2010). The interaction between a target protein and small molecules is one of the most studied subjects in the field of drug discovery (Hughes et al. 2011). Many strategies have been devised to effectively screen the small molecules that bind the protein of interest, which includes structure-based virtual screening, high throughput screening, and cell-based screening (Lionta et al. 2014; Carnero and Carnero 2006; Nierode et al. 2016). In the field of high throughput screening, the NMR spectroscopy has its own position due to its capacity to monitor the dynamic process of binding, which shows the molecule that binds the target protein or what part of the protein is in contact with the ligand (Skinner et al. 2008). The WaterLOGSY would be the popular version of the saturation-transfer difference (STD) experiment to study the interaction between the protein and the ligand (Raingeval et al. 2019; Antanasijevic et al. 2014). It can identify the binding ligand, and determine the binding constant (Huang et al. 2017). This type of method relies on NMR experimentation to observe the intensity changes. We wished to take a different path: to prepare an alternative material that will produce the desired data with a less sophisticated NMR method.

We chose the elastin-like polypeptide (ELP) as the module that could provide the desired property to our material. It was derived from elastin, which is an essential protein of the extracellular matrix (Le et al. 2019). The ELP is composed of pentapeptide repeats (VPGXG)_n_ where X can be any amino acid, and it tends to reversibly aggregate above the transition temperature (Despanie et al. 2016). Depending on the number of repetitions and/or the identity of X, the transition temperature varies a lot (Christensen et al. 2013; Teeuwen et al. 2009). In general, the transition temperature becomes lower with a higher repetition number and/or a larger hydrophobicity of X (Kowalczyk et al. 2014). This ELP can be fused to a protein of interest, and the fusion protein retains the temperature-dependent characteristics of the ELP as well as the specific property of the target protein (Christensen et al. 2013). This property can be applied to an alternative strategy for protein purification where the slightly elevated temperatures are used to precipitate the fusion protein without denaturation (Simnick et al. 2007).

While others tried to overcome or reduce the line broadening to observe the signals better, we tried to take advantage of it to better remove the signals of interest from the spectrum. Our method essentially depends on the relaxation process and its effect on the line broadening. We pushed this broadening to the extreme, that is, until the signal vanishes. The ligands are generally small molecules, and their signals are very sharp compared to those from the proteins. We found a method to make the protein very large by using ELP, so it might be treated as a solid surface with many binding sites because it would not tumble in the solution in a practical sense. If the ligand binds the protein in this type of state for a sufficient amount of time, it should then also become a non-tumbling mass and would lose its magnetization or signal. Therefore, we can identify the binding ligand from a mixture of compounds by just looking for a molecule that loses its signal. We believe that our method offers an easier method for this type of identification than the current methods because the latter is based on the reduced signal intensity and/or the changed chemical shift and/or more sophisticated NMR experimentation. We expect that this will be extended to the two-dimensional spectroscopy if we wanted to explore a mixture of many compounds, so the overlap should be managed. This method has been submitted for a Korean patent application, which is number 10-2018-0123555.

## Materials and methods

### Plasmid construction

Plasmid pVP65K was kindly provided by Dr. Ronnie O. Frederick (University of Wisconsin – Madison, Madison, WI, USA). The plasmid (I48 ELP) that contained the elastin-like polypeptide gene was purchased from Addgene (Watertown, MA, USA). The construction of the pVP65KR will be reported elsewhere. The gene block that corresponds to the maltose binding protein (MBP) fused to mCherry fused to octahistidine tag (MBP-mCherry-8xHis) polypeptide was synthesized by Integrated DNA Technologies, Inc (Coralville, IA, USA). This gene block did not have any inside stop codon, and all three components were in the same reading frame. The restriction sites, which included the NcoI and NotI, were created by the PCR at the 5′ and 3′ ends. The pVP65K and this gene block were digested with the NcoI and NotI enzymes and then ligated. The resulting plasmid was named the pVP65KR. The MBP gene without a stop codon was amplified from the pVP65K by the PCR with the SgfI and PmeI sites at the 5′ and 3′ ends. The vector pVP65KR and this MBP gene were digested with the SgfI and PmeI enzymes and then ligated. The resulting plasmid was named the pVP65KR-MBP. The I48 gene was modified to create a SacI site at the 5′ end using the QuikChange Lightning Site-Directed Mutagenesis Kit (Agilent, Santa Clara, CA, USA). The I48 ELP was digested with the SacI and HindIII enzymes, purified, and ligated with the pVP65KR-MBP, which was digested with the same enzymes. The resulting plasmid was named pVP65KR-MBP-I48 (Fig. S6). The strain DH5α (RBC Bioscience Corp., New Taipei City, Taiwan) was employed in all the plasmid construction works. As a control, the I48 ELP gene was prepared from the original plasmid by the restriction digestion with the NdeI and SalI. It was ligated with the pET28a(+), which was previously digested with the NdeI and XhoI. The resulting plasmid was named the pET28a-I48ELP.

### Protein preparation

The plasmid, which includes the pVP65KR-MBP and the pVP65KR-MBP-I48, were brought into the Rosetta2(DE3)pLysS (‎Merck-Millipore, Burlington, MA, USA). A single colony was used to inoculate a 100 mL Overnight Express™ Instant LB Medium (‎Merck-Millipore, Burlington, MA, USA), which was grown for 24 h, harvested by centrifugation at 4000 rpm for 15 min at 4 ℃, resuspended in 10 mL of 10 mM TrisHCl pH 8.2, and stored at − 20 ℃. The frozen cells were thawed, 1 mg of DNaseI (Sigma-Aldrich Corp., St. Louis. MO, USA) was added and incubated for 10 min, RT. Triton X-100 (Sigma-Aldrich Corp., St. Louis. MO, USA) was added to the final concentration of 1%, and the supernatant was retained by centrifugation at 17,000 rpm for 30 min at 4 ℃. The supernatant was loaded onto a 5 mL HisTrap™ Fast Flow column (GE Healthcare Life Sciences, Pittsburgh, PA, USA) that was installed on a BioLogic LP System (Bio-Rad, Hercules, CA, USA). The column was washed with 10 column volumes of an NPI-10 buffer, which included 50 mM sodium phosphate pH 7.0, 300 mM NaCl, and 10 mM imidazole, and it was applied to a linear gradient to 50% NPI-500, which included 50 mM sodium phosphate pH 7.0, 300 mM NaCl, and 500 mM imidazole, over 10 column volumes. The fractions that contained the MBP or the MBP-I48 were pooled, concentrated, and buffer-exchanged to 10 mM sodium phosphate pH7.0 by ultrafiltration (Vivaspin® 20, Sartorius, Goettingen, Germany). The concentrated protein solution was quantified using the ProtParam module of the ExPASy server (https://web.expasy.org/protparam/) (Gasteiger et al. 2005) and lyophilized. The I48 ELP was prepared using a similar method.

#### NMR experiments and data processing

All the NMR samples for the MBP-I48 contained 10 mM sodium phosphate pH 7.0, 0.5 mM DSS, which contained 4,4-dimethyl-4-silapentane-1-sulfonic acid, and 0.5 mM DFTMP, which contained 1,1-difluoro-1-trimethylsilanyl methylphosphonic acid, in 600 µL of D_2_O. The ligand concentration was fixed at 50 µM, and the protein concentrations were 0.3, 1.0, 5.0, 15, and 50 µM for maltose, or 0.15, 0.5, 1.5, 5.0, 15, and 50 µM for all other sugars. The samples for the MBP and the I48 ELP contained 10 mM sodium phosphate pH 7.0 and 0.5 mM DSS. Also, the concentrations for this polypeptide were 0.15, 0.5, 1.5, 5.0, 15, and 50 µM, and the maltose concentrations were set to 50 µM. All the experiments were performed using Bruker Avance II 500 MHz equipped with a TXI probe. A one-dimensional version of NOESY pulse sequence (noesypr1d) was employed. The ^1^ H spectra were collected with 48 K data points over the spectral width of 12 ppm. The residual water resonance was suppressed by presaturation. The NOESY mixing time was set at 50 ms, and 128 transients were collected per experiment. The raw data was apodized by an exponential window function with a line broadening factor of 0.5 Hz, zero-filled to 64 K, Fourier transformed, and phase adjusted with Mnova NMR (Mestrelab Research, S.L., Santiago de Compostela, Spain). The DSS resonance was used to reference the chemical shift.

## Results and discussion

### Concept

**Fig. 1:**
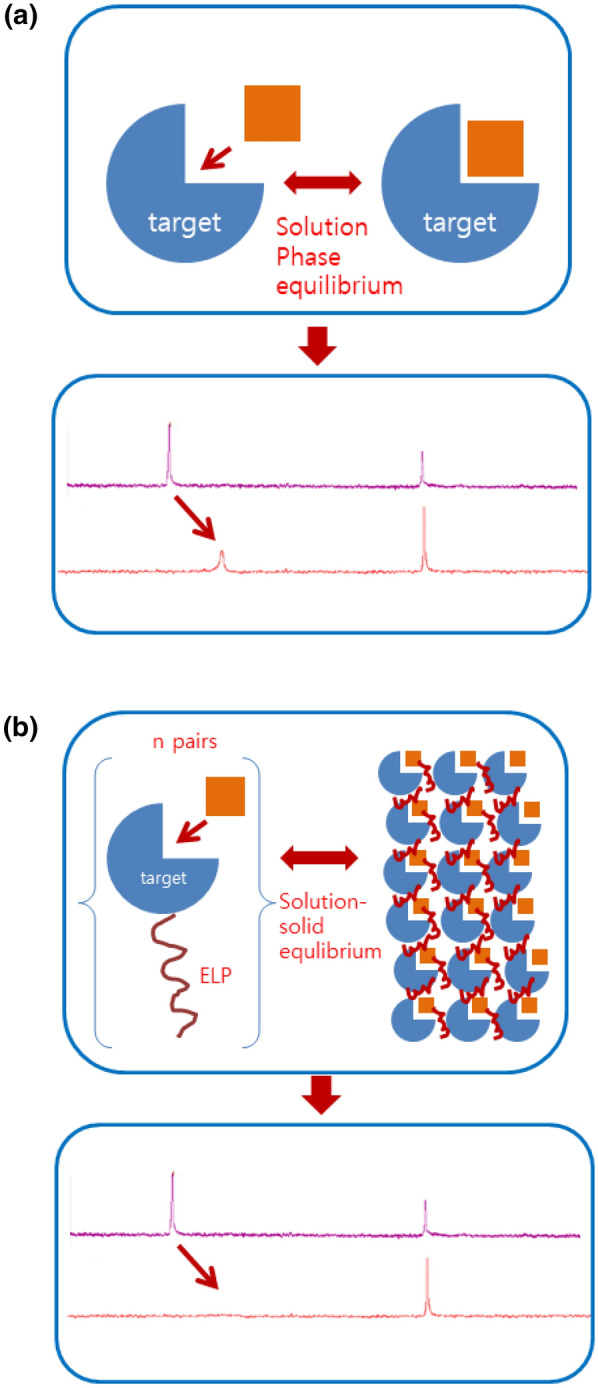
A conceptual diagram of our method. **a** The traditional ligand screening, and its change in the spectrum. **b** The proposed ligand screening, and its change in the spectrum

We intended to develop an alternative NMR-based method to screen the small molecules that could bind a target protein. The NMR provides information that is sensitive to the environment of the ligand, and most methods or observations are based on the movement and/or the reduction of the ligand signal, which is illustrated in Fig. 1a (Skinner et al. 2008; Maity and Gundampati 2019; Becker et al. 2018). However, this kind of change may not be large enough to be discerned. While devising a method to amplify this change, we came up with an idea that immobilizing the ligand would lead to the disappearance of its NMR signal due to the extreme line broadening. The signal broadening is something that all the solution NMR spectroscopists hope to avoid, but it is indeed necessary for erasing the signal of a bound ligand in our case. To erase the signal of a ligand, we have to make sure that the ligand can not only be bound to a receptor and also the receptor-ligand complex should be a non-tumbling or very slowly tumbling mass, which means that the receptor should be either very large or be solid support. This signal disappearance would be recognized more readily and sensitively than the shift or reduction, which is shown in Fig. 1b. In fact, Zhang et al. used the charged nanoparticles to simplify the spectrum and facilitate the metabolite identification (Zhang et al. 2015). The size of these types of nanoparticles was large enough to tumble/rotate very slowly in the solution. They used a two-dimensional NMR method, which included the (HSQC and the heteronuclear single quantum coherence, and observed that the cationic or anionic metabolites lost their signals when they were mixed with oppositely charged nanoparticles because the tumbling rate of the metabolite also became very slow one captured so that their magnetization got dissipated quickly. The charged nanoparticles acted like chromatographic beads, and the oppositely charged compounds seemed to stay on them quite stably. Any molecule with a negatively or a positively charged surface can bind to the nanoparticles of opposite charges, which is like ion-exchange chromatography. The affinity version of this kind was also reported under the name of TINS (Target Immobilized NMR Screening) (Vanwetswinkel et al. 2005). This technique is based on the immobilized target protein which is chemically coupled to the sepharose resin. The detection strategy relies on the difference between two spectra which are recorded with or without the resin.

We wished to study the specific interaction between the ligand and the receptor while avoiding the preparation work involved in the chromatographic materials. We also wished to simplify the detection: the disappearance of the signal should be better than its change. We went on to devise a very large biomaterial with an affinity to the specific molecule. The elastin-like polypeptide (ELP) was chosen to serve this type of purpose, because (1) it could be produced in the *E. coli* in a recombinant fashion with the target receptor attached, (2) it could undergo a reversible transition between the monomers and the supramolecular aggregates, and (3) the attached receptor could remain intact, which means it retains its binding ability, in the aggregates that could be regarded as a very large mass with many ligand binding sites. In summary, our system was designed to act as an affinity chromatography system, because only the molecules with a specific binding ability could be adsorbed into the solid support. Also, what we needed to identify the binding ligand was to look for the signals that disappeared when the mixtures were mixed with the receptor aggregates.

### Plasmid construction and protein preparation

The construction of a plasmid is usually straightforward, which included amplification of a gene of interest that is followed by digestion with suitable restriction enzymes, which is followed by ligation with the vector of choice. However, the highly repetitive nature of the ELP sequence hindered successful amplification, which is the very first stage of the entire process. In the present case, we have 48 repetitions for the Val–Pro–Gly–Ile–Gly, which corresponds to 5′-gttccgggcatcggt-3′, which means just a 15 bp sequence (Shi et al. 2013). Positioning two primers at both flanking regions resulted only in a ladder of bands. This type of ladder formation has been observed for other repetitive sequences, even though the length of this type of repetitive sequence was about 100 bp, which is much longer than ours (Riet et al. 2017). To prevent this type of PCR artifact, Tang and Chilkoti employed codon scrambling, which basically utilized the 3′ wobbling or codon degeneracy (Tang et al. 2016). Instead of going through this seemingly complicated process, we chose to create suitable restriction sites by site-directed mutagenesis and to cut out the sequence. Even though the mutagenesis procedure was based on PCR-like amplification, the repetitive region was preserved, which was confirmed by the DNA sequencing, not diverging into a ladder. This is actually what Hommelsheim et al. observed. If two primers were set distantly from the repetitive region, a lesser ladder was formed (Hommelsheim et al. 2014). In the case of the mutagenesis, the two primers were annealed at the same position but on different strands, and one strand was synthesized in the opposite direction of the other, which made the effective distance between those two primers very long. This means that the newly synthesized strand met the other only after making a full circle of the plasmid. A detailed and systematic study on this ladder formation of our repetitive sequence will be reported elsewhere. The final construct could produce the MBP fused to the mCherry fused to the octahistidine tag fused to the MBP fused to the I48. The first MBP was the solubilization tag, whereas the second MBP was the actual test target protein. The other target proteins can be substituted for this MBP. The mCherry functions as a reporter of the target protein production, and the whole cell culture turned red if the induction of protein production was successful. While we were developing this plasmid, Hui et al. reported a similar vector independently (Hui et al. 2018). The construction of our version of the universal vector system will be reported elsewhere. The MBP-I48 was cleaved intracellularly by the TVMV protease produced at a basal level by the same plasmid, which occurred even before the cells were harvested and lysed (Blommel et al. 2009; Aceti et al. 2015). Since the MBP-I48 had an octahistidine tag at the N-terminal end, it was purified using simple affinity chromatography with an imidazole gradient. The buffer was exchanged to a 10 mM phosphate buffer, concentrated, and lyophilized, which was absolutely necessary for the NMR experiments where a deuterated solvent was used.

### NMR experiments and data interpretation

All the NMR samples for the MBP-I48 contained 0.5 mM DSS for the chemical shift reference and 0.5 mM DFTMP for the pH monitoring (Reily et al. 2006)of the samples for the I48-ELP did not contain the DFTMP. The resonances at 0, 0.63, 1.76, and 2.91 ppm belong to the DSS. The single resonance at 0.19 ppm belongs to the DFTMP. All the NMR experiments were performed according to the recommendation from Chenomx NMR Suite (Chenomx Inc, Edmonton, AB, Canada) to use its database and perform the quantitative analysis. The 1D NOESY pulse sequence was used to selectively irradiate the water resonance during the relaxation delay, which was the delay between the scans, and also during the mixing time (Ross et al. 2007). The water signal can be suppressed if necessary by the Mnova processing software. We focused on the peaks that originated from the anomeric protons that were located between 4.0 and 5.5 ppm. These peaks were outside the crowded region, and their intensity changed upon binding to the protein was easily monitored. The maltose peaks are located at 5.2 and 5.4 ppm, and the pattern is clearly different from the glucose, lactose, or galactose that does not have a peak at 5.4 ppm. The lactose had one additional peak at 4.4 ppm, and the galactose had one additional peak at 4.6 ppm, which act as an additional handle for comparison. The sucrose has a peak at 5.4 ppm, which almost overlaps with the maltose peak. However, it also has additional peaks at 4.1 and 4.2 ppm. Therefore, we can distinguish the maltose from the other sugars even in a mixture by the presence of the peak at 5.4 ppm and by the absence of the peak below 5.0 ppm in the anomeric proton region. We fixed the concentration of the sugar at 50 µM and increased that the concentration of the MBP-I48 step wisely to observe the spectral changes. The concentrations of the protein were 0.15, 0.5, 1.5, 5, 15, and 50 µM, which covered the protein-to-ligand ratio from 1:333 to 1:1 (excess of ligand). As a negative control, the nonspecific binding for the maltose to I48 ELP was tested, and the NMR spectra showed that the maltose signal remained the same for all the concentrations of the I48 ELP at either 288 or 298 K, which is shown in Fig. S3. As expected, the I48 ELP also showed a temperature-dependent aggregation, so there were fewer and weaker signals at 298 K than at 288 K. As a positive control, the binding of the maltose to the MBP was tested, and the trend looked similar to the trend for the MBP-I48, which the maltose signal diminished as more MBP was added. This is illustrated in Fig. S4. Since the MBP remained monomeric at 298 K, the protein signals were visible. If a higher concentration had been used, they might have obscured the maltose signals.

**Fig. 2:**
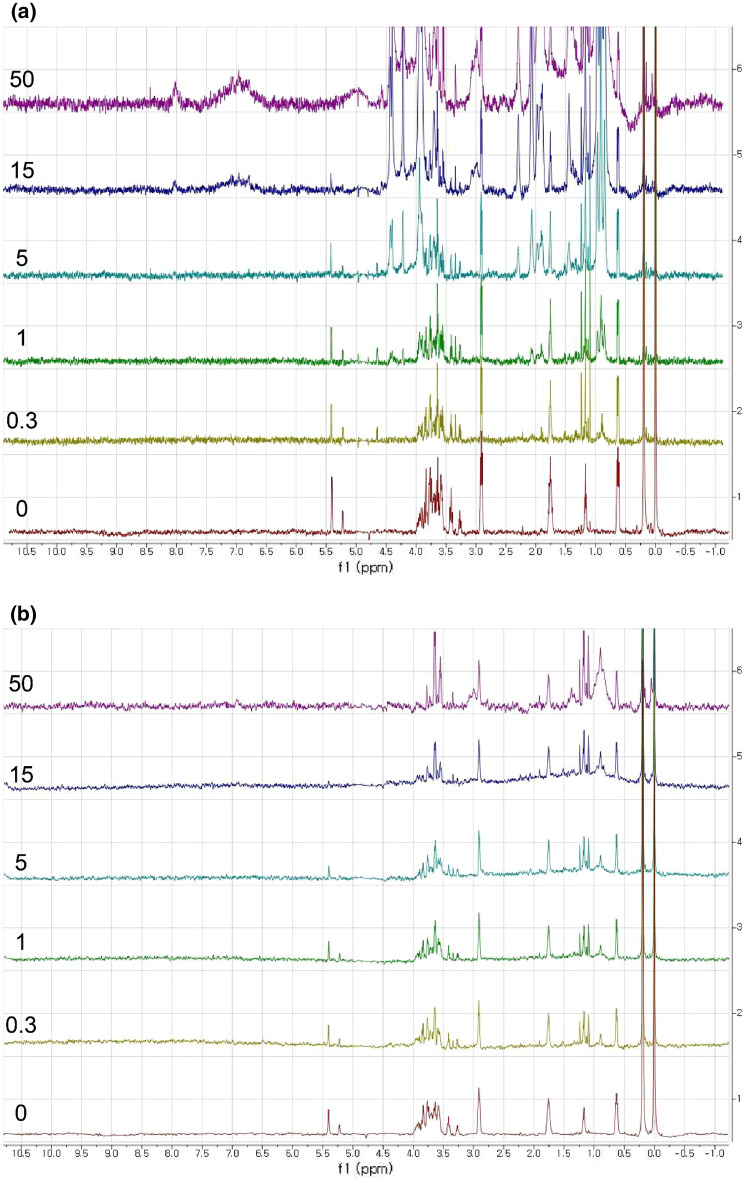
A stacked plot of the H-1 spectra of the mixture of the MBP-I48 and maltose. The trace corresponds to the protein concentrations of 0, 0.3, 1.0, 5.0, 15, and 50 µM, which is from the bottom, collected at **a** 288 K and **b** 298 K. The traces are labeled with protein concentration in µM

The effect of the protein aggregation on the reduction or the complete disappearance of the maltose signal could be monitored by elevating the temperature. Figure 2 shows a stacked plot of the spectra collected at 288 K (a) and 298 K (b) with varying protein-to-ligand ratios. The protein signals became visible above the concentration of 5 µM at 288 K, which was well below the reported transition temperature of the aggregation of I48 ELP and 295 K (Shi et al. 2013). Even though the transition temperature of the fusion protein may differ from this value, it seems that the transition occurred between 288 and 298 K based on the following observation. If a spectrum at 298 K, which is shown in Fig. 2b, is compared to the corresponding one at 288 K, which is shown in Fig. 2a, we can see that most of the signals have disappeared except for the fast-rotating methyl groups around 0.9 ppm. We believe that the sharp peaks around 1.1 ppm and 3.6 ppm originated from the impurities in the protein sample, so the protein peaks should be much broader than the peaks of the small molecules. Thus, we can say that the MBP-I48 is aggregated at 298 K but not at 288 K, which implied that the temperature of 298 K was near or above the transition temperature. We preferred using a temperature of 298 K, because it was regarded as the standard. Also, we wished to keep the protein intact, because higher temperatures could have damaged the protein. We could also observe that the maltose signals disappeared approximately at the protein-to-ligand ratio between 3:1 and 1:1 at 298 or 288 K. The molecular weight of the MBP-I48 was calculated to be 63.4 kDa, which was large enough to broaden the signal of the bound maltose extensively. We also tried the MBP as a positive control in order to show that the binding affinity did not change as it was fused to the I48 ELP, which is illustrated in Fig. S4. The molecular weight of the prepared MBP was calculated to be 44.3 kDa, which included the 8xHis tag and the residues from the multiple cloning sites. It was smaller than the monomeric MBP-I48, so more signals were clearly visible at the concentration of 50 µM. As expected, the maltose signal was reduced as more MBP was added. The advantage of using aggregation is that we could make the protein signals disappear, and it became easier to observe the ligand signals, of which there were no background signals from the protein. In other words, aggregation is a desirable way to increase the apparent size of the protein, while maintaining the native state of the protein. In this test case, the aggregates of the MBP-I48 are analogous to the chromatographic beads coated with the MBP. As the charged nanobeads effectively eliminated the signals from the bound compounds, which were oppositely charged, the aggregated receptor could do the same to the bound ligand by making it tumble very slowly, which is illustrated in Fig. 2b.

### Competition test

**Fig. 3:**
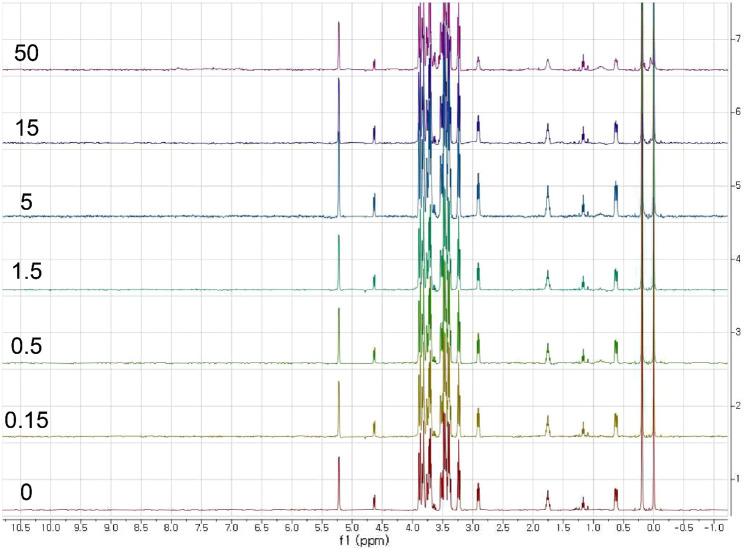
A stacked plot of the H-1 spectra of the mixture of the MBP-I48 and glucose. The trace corresponds to the protein concentrations of 0, 0.15, 0.5, 1.5, 5, 15, and 50 µM, which is from the bottom, collected at 298 K. The traces are labeled with protein concentration in µM

The binding or the selection ability of the MBP for the maltose against several sugars was tested, which included glucose, lactose, galactose, and sucrose. Glucose was chosen, because it is the building block for maltose, and we thought it would at least loosely fit the binding pocket. Both lactose and sucrose were chosen, because they contained the glucose moiety, and we thought that they might get loosely hooked by the binding pocket. Galactose was chosen as a negative control. The result was that none of them were bound to the protein, and their signals did not change with the proteins that were added, which is illustrated in Figs. 3 and S1. In fact, there was an X-ray structure report that illustrated that the binding of the maltose to the MBP should only be driven by the interaction between the α(1–4) linkage portion and the binding pocket, which was interpreted as the cooperative hydrogen bonds (Quiocho et al. 1997). Even though the result negative, this result provided integrity of our system. We also tested the maltotriose and β-cyclodextrin, which will be discussed in the next section.

**Fig. 4:**
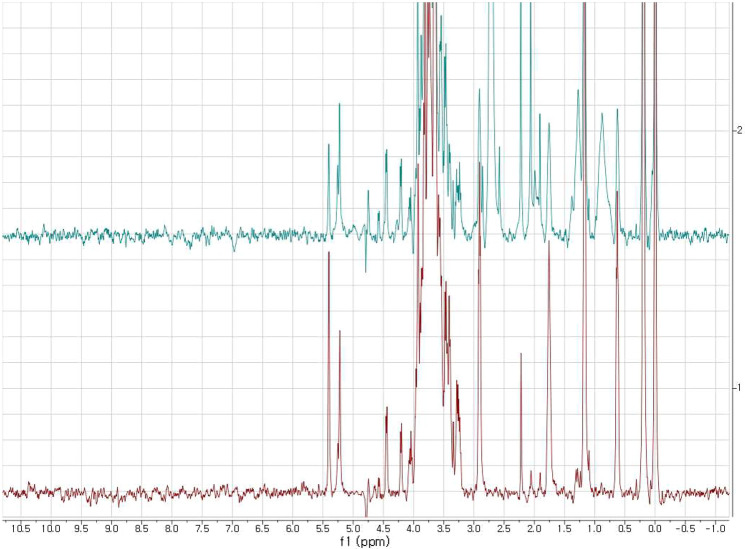
A stacked plot of the H-1 spectra of the mixture of the MBP-I48 and the selected sugars. The trace corresponds to the sugar mixture only, which is at the bottom, and the sugar mixture plus the protein, which is at the top, at 298 K

To show that our system could pick up the maltose from other sugars, we performed a competition experiment by making a mixture of the sugars mentioned above, which include maltose, glucose, sucrose, lactose, and galactose. Again, most of the background protein signals vanished at 298 K, which obscured ligand signals at 4.4 and 4.2 ppm at 283 K. According to the spectra, maltose was the only one that could actually bind the MBP, so its signal was the only one that vanished, which is illustrated in Figs. 4 and S2. The two key signals for the maltose were located at 5.40 and 5.23 ppm, which were overlapped with of the signals for the sucrose and glucose. Both the signals were reduced with the addition of the MBP-I48 at 298 K. We already showed that neither the glucose nor the sucrose was bound to the protein, which led to the conclusion that the signal reduction in Fig. 4 was due to the disappearance of the maltose signals. The signals of the other sugars remained intact. This competition test was to demonstrate the utility of our method, because it clearly showed that it was able to tell the sugars that could or couldn’t bind to the protein in a mixture of the molecules, so the binding does not have to be tested one molecule at a time. Nevertheless, even this simple mixture resulted in some overlap, which might suggest the necessity of two dimensional or higher NMR experiments, which would become a requirement when simultaneously screening many compounds.

### Siblings test

**Fig. 5:**
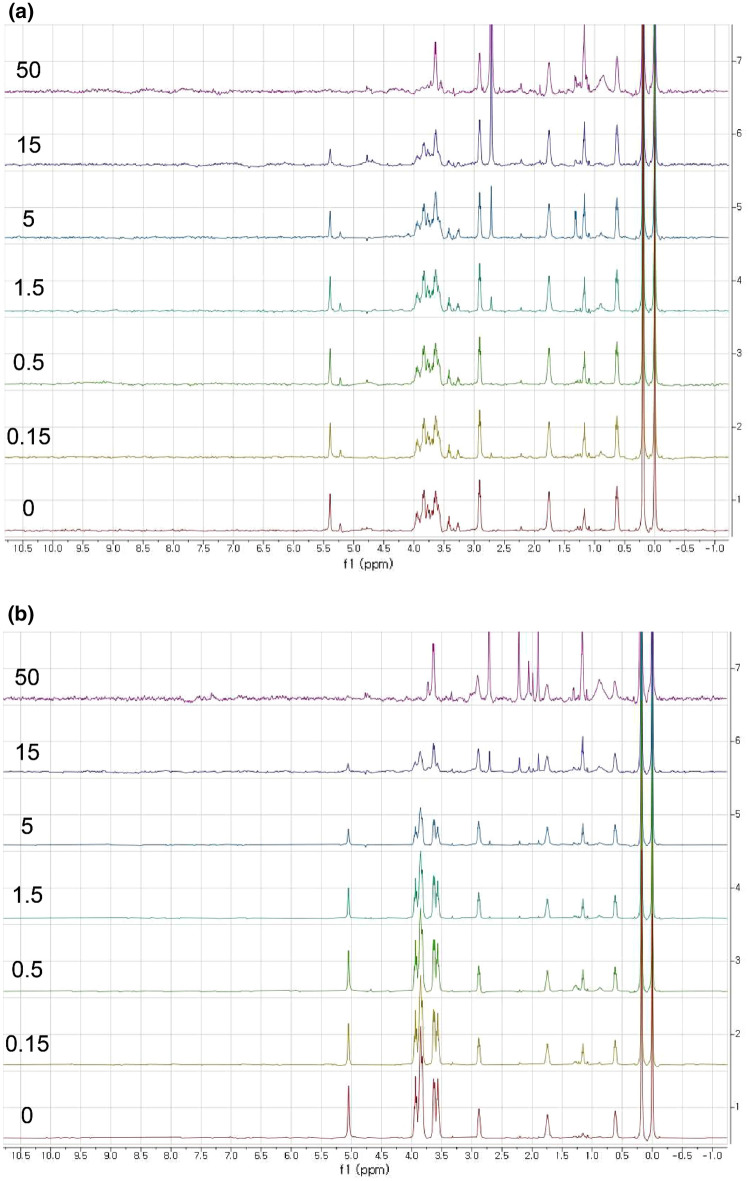
A stacked plot of the H-1 spectra of the mixture of MBP-I48, **a** maltotriose, and **b** β-cyclodextrin. The trace corresponds to the protein concentrations of 0, 0.15, 0.5, 1.5, 5, 15, and 50 µM, which is from the bottom, collected at 298 K. The traces are labeled with protein concentration in µM

The maltose, maltotriose, and β-cyclodextrin have been reported to bind the MBP, and the known dissociation constants are 3.5, 0.16, and 1.8 µM (Quiocho et al. 1997; Sharff et al. 1993). As expected, the signals from these two molecules disappeared at the protein-to-ligand ratio between 1:1 and 1:3, but it does not look that the disappearing rates were different, which is illustrated in Figs. 5 and S5. We are currently working on more quantitative analyses to see if there would be any correlation between the rate of the signal disappearance and the dissociation constant.

In addition to the WaterLOGSY (Raingeval et al. 2019; Antanasijevic et al. 2014; Huang et al. 2017), STD NMR(Viegas et al. 2011), and TINS (Vanwetswinkel et al. 2005) mentioned earlier, there are many other NMR techniques for ligand screening. The basis of their characteristic detection method can be roughly classified into 3 categories: the line broadening, the chemical shift perturbation, or the magnetization transfer. To detect the line broadening effectively, the difference of the molecular sizes between the ligand-bound and ligand-free state has to be large. The methods, for example, AIDA-NMR (Rothweiler et al. 2008)or FAST-NMR (Mercier et al. 2006), try to detect a spectral change stemming from the different molecular sizes. On the other hand, our method utilizes the extreme case, and it can provide the maximum possible difference. To observe the chemical shift perturbation, one has to overcome the overlap problem, and the isotopic labeling, either specific (3-FABS (Dalvit et al. 2004) or RAMPED-UP NMR (Zartler et al. 2003)) or uniform (SAR by NMR (Shuker et al. 1996)) of the target protein, is almost obligatory. As stressed earlier, our method removes the signals of the binding ligands as well as the background signals from the proteins, and we believe that it should be easier to observe the difference. The STD utilizes the signal enhancement of the ligand-bound state, and the resulting spectra can be viewed as the mirror image of the line broadening technique. It depends on the different enhancement between the free and bound state, and the degree of enhancement is limited like the line-broadening technique. Our method maximizes the difference by effectively removing the signals of the bound state, and it can be regarded as an on/off type of observation which should offer a clearer comparison than the reported methods such as multi-step NMR (Mercier et al. 2009), INPHARMA (Sánchez-Pedregal et al. 2005), NOE pumping (Chen et al. 1998), or SALMON (Ludwig et al. 2008) could provide.

Like all other detection systems, our method can also produce false positives and/or false negatives. The false positives/negatives would mostly originate from the ELP module itself and/or the linker between the target and the ELP. The non-specific binding to the target protein can be regarded as a true positive in our interest because our strategy is to tether such weak binders to assemble a potent candidate. As shown in the report, a control experiment with the ELP module alone can sort out possible false positives. The linker can be a source of false positives as well as false negatives. If the linker is long enough, it can give freedom to the target and the ELP to act independently, but there is a possibility that it assumes a conformation that can bind to some molecules, resulting in false positives. Or, the ELP can interact with the target protein, masking the binding site of a ligand, which would yield a false negative. However, we also have the interactions among ELPs to form aggregates, and this interaction is quite stable above the transition temperature, which may open up the masked binding site of the target protein by pulling away from the ELP module. Then, the aggregation would be beneficial for the ligand binding and reduce this type of false negatives. On the other hand, if the linker is too short, then the ELP can restrict the movement of the target or vice versa, which may prevent the formation of the desired aggregates, resulting in less sensitive detection.

Our method can be applied to many other proteins/receptors. The proper expression of the target proteins fused with the ELP should be the first major step to make. If the target protein is large enough, the bound ligand may then vanish without the ELP moiety. However, even with the size of the MBP or the MBP-I48, the background protein signals still remained. The protein signals were effectively suppressed when the target protein formed aggregates, which will provide an easier way to inspect the entire range of the spectrum in order to detect the binding ligands. The real power of this method would be manifested if the target protein is small enough that the signal of the binding ligand only gets broadened to an extent. If a small protein is fused with the ELP, the binding ligand would then completely lose its signal above the transition temperature, which should facilitate the analysis. Another large advantage of this method would be the high throughput screening with the mixture of the candidate compounds because only the binders’ signals will be lost from the spectrum. According to Bruschweiler group’s result, the signal reduction, which is due to the binding to the charged resin depended on the opposite charges of the small molecules, the degree of signal reduction of the histidine was less than that of the lysine or the arginine. In other words, if a compound stayed for a shorter time, the signal reduction was then decreased. We expect that our system will show a similar result, but it will also lead to finding weak binders. We think that we can increase the protein concentration in order to pick up those molecules in a clean background. We can suppress the protein signals by slightly elevating the temperature. Detailed analysis of the kinetic and thermodynamic parameters is underway. On the other hand, a one-dimensional spectrum will inevitably suffer from the peak overlap, and we think that a two-dimensional experiment would be necessary if there are many components in a mixture. If the ligands are labeled with carbon-13 or nitrogen-15, we can then perform a two-dimensional HSQC experiment, which will show a very limited number of resonances compared to the homonuclear one-dimensional or two-dimensional experiments, which will again be facilitating the analysis or the detection. In this isotope-assisted experiment, the protein concentration could be very much increased. We have the experience to prepare the pool of C-13 labeled metabolites by growing *E. coli* in a minimal medium with the C-13 enriched glucose as a sole carbon source. The N-15 labeling would be conducted by growing them in a medium with the N-15 enriched ammonium chloride or ammonium sulfate. These types of experiments are now underway with several pairs of receptors and ligands, and we hope that the results will come out as expected.

The world has been suffering from the COVID-19 pandemic for more than a year, and it has been predicted that these types of pandemics will keep coming in the future. In order to be able to cope with these types of viral outbreaks, a new drug will be needed that is more effective than a vaccine. We hope this method can help in some way to the development of the first round of binders, which would lead to potent candidates.

## Supplementary Information

Below is the link to the electronic supplementary material.

Supplementary material 1 (DOCX 2102.4 kb)

## Data Availability

The raw and processed data in this work will be available from Young Kee Chae upon request.
