# Supplementary material for: A simple and sensitive detection of the binding ligands by using the receptor aggregation and NMR spectroscopy: a test case of the maltose binding protein
Source: J Biomol NMR. 2021 Sep 15;75(10-12):371–81. doi: 10.1007/s10858-021-00381-x (PMC8441238; doi:10.1007/s10858-021-00381-x)

Figure captions

Figure S1. Stacked plot of the H-1 spectra of the mixture of MBP-I48 and (a) sucrose, (b) lactose, and (c) galactose. The trace corresponds to the protein concentration of 0, 0.15, 0.5, 1.5, 5, 15, or 50 μM (from the bottom) collected at 298 K. The traces are labeled with protein concentration in μM.

Figure S2. Stacked plot of the H-1 spectra of the mixture of MBP-I48 and selected sugars. The trace corresponds to the sugar mixture only (bottom) and sugar mixture plus the protein (top) at 298 K. The expanded region for sugar monitoring is shown. The traces are labeled with protein concentration in μM.

Figure S3. Stacked plot of the H-1 spectra of the mixture of I48-ELP and maltose. The trace corresponds to the protein concentration of 0, 0.15, 0.5, 1.5, 5, 15, or 50 μM (from the bottom) collected at (a) 288 K and (b) 298K. The top trace corresponds to I48-ELP with no maltose. The traces are labeled with protein concentration in μM.

Figure S4. Stacked plot of the H-1 spectra of the mixture of MBP and maltose. The trace corresponds to the protein concentration of 0, 0.3, 1, 5, 15, or 50 μM (from the bottom) collected at 298K.

Figure S5. Relative intensity changes of maltose, maltotriose, and β-cyclodextrin with respect to the added MBP-I48 or that of maltose with added MBP.

Figure S6. The plasmid map of pVP65KR-MBP-I48.

Figure S1(a)


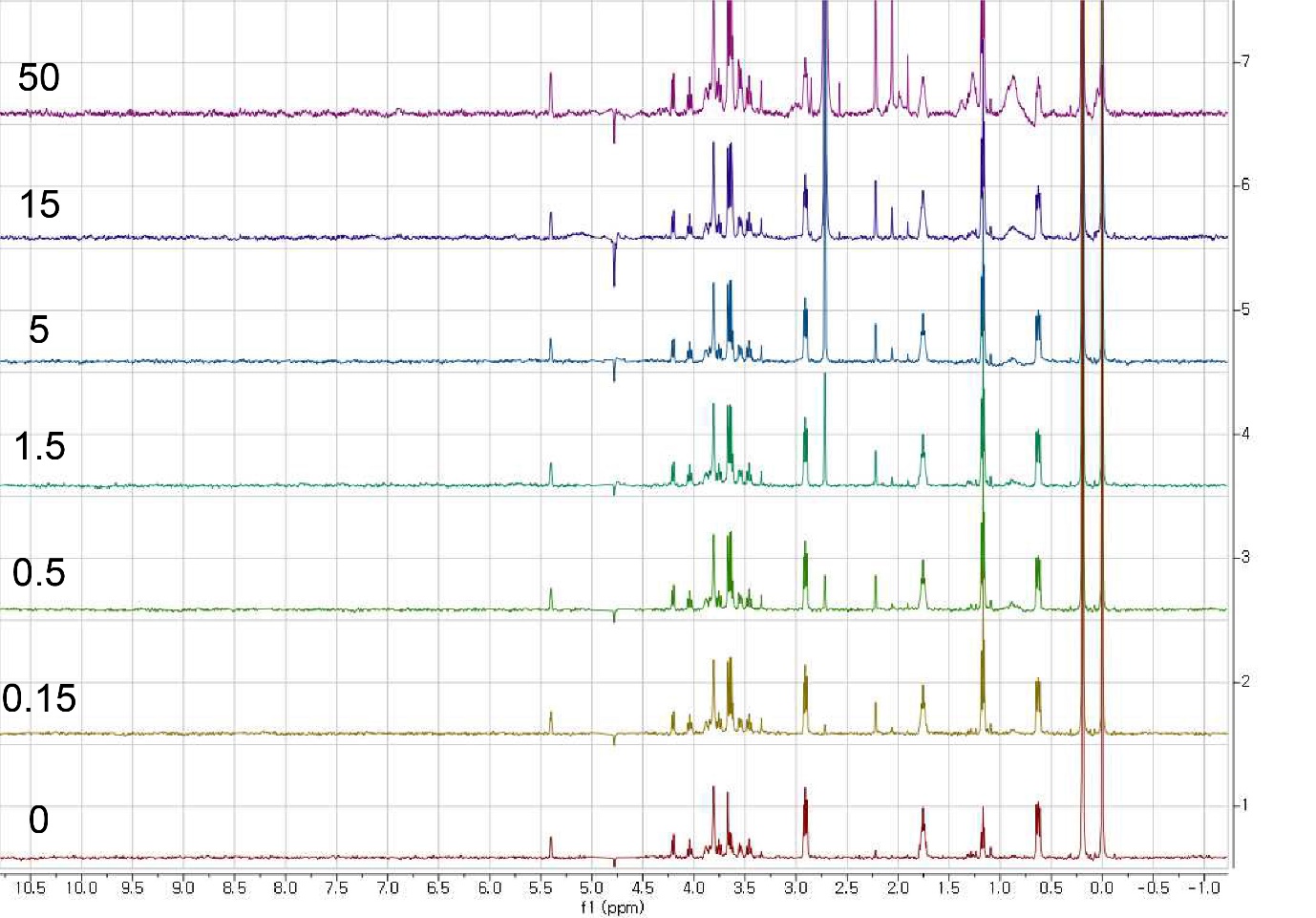


Figure S1(b)


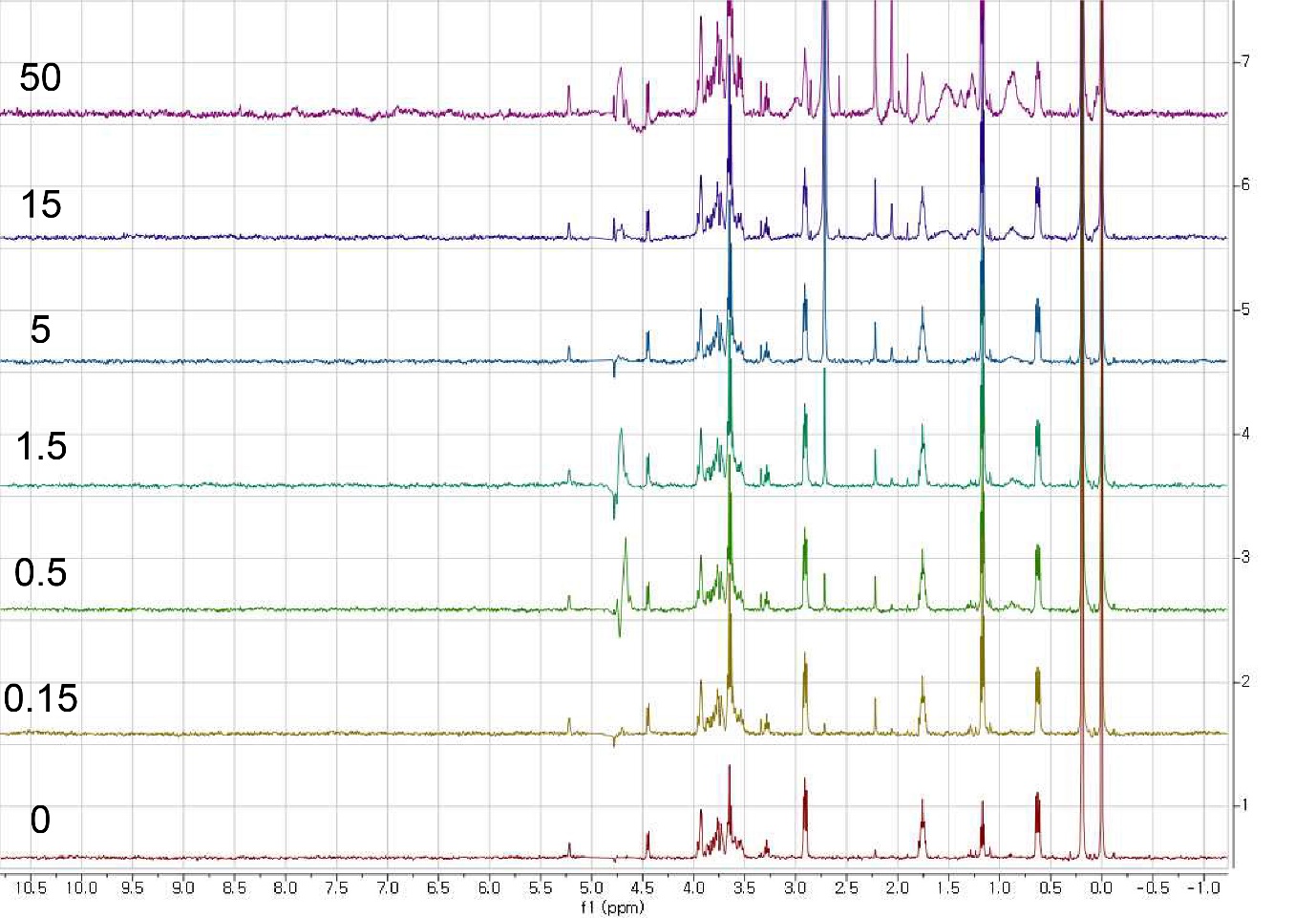


Figure S1(c)


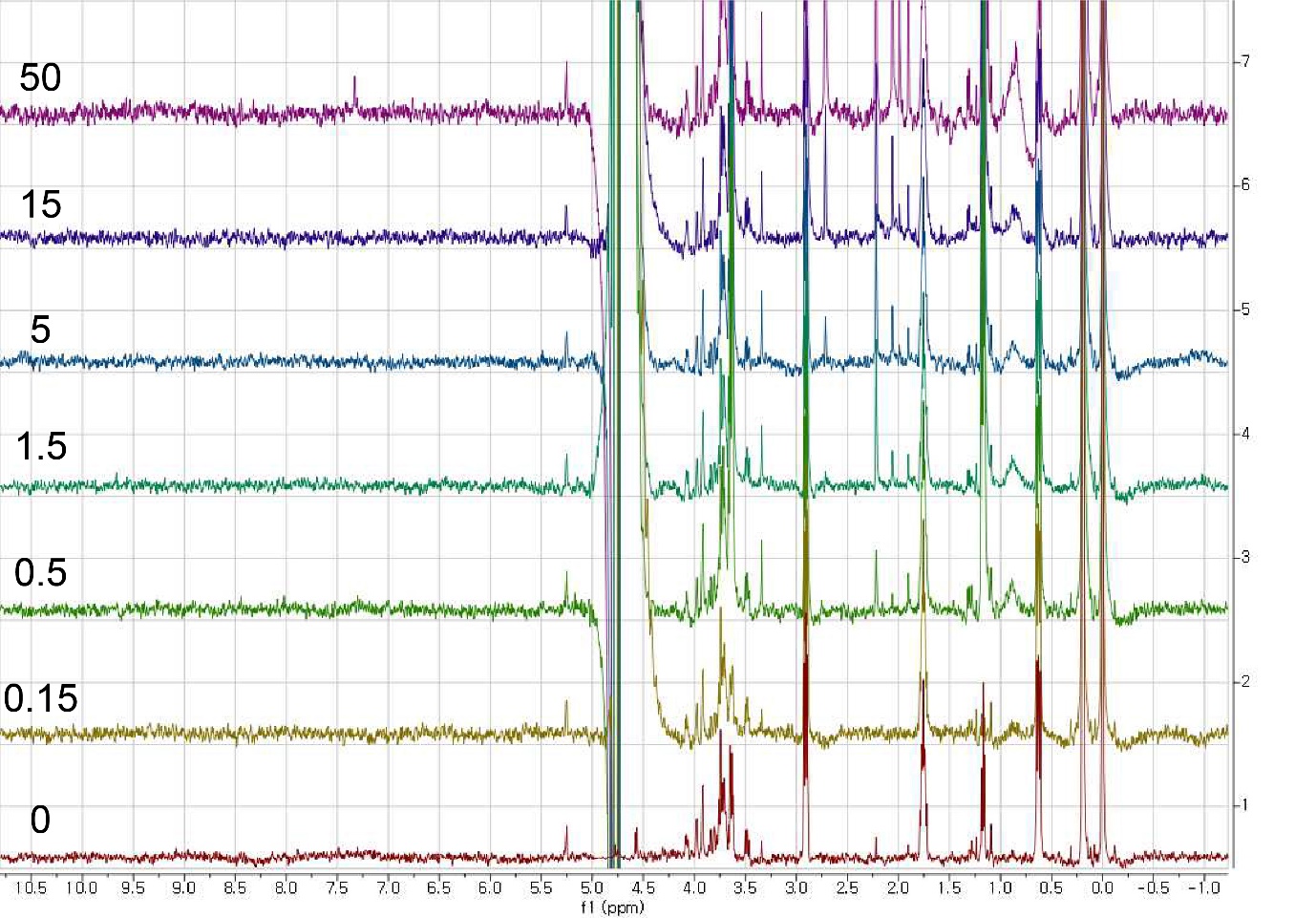


Figure S2


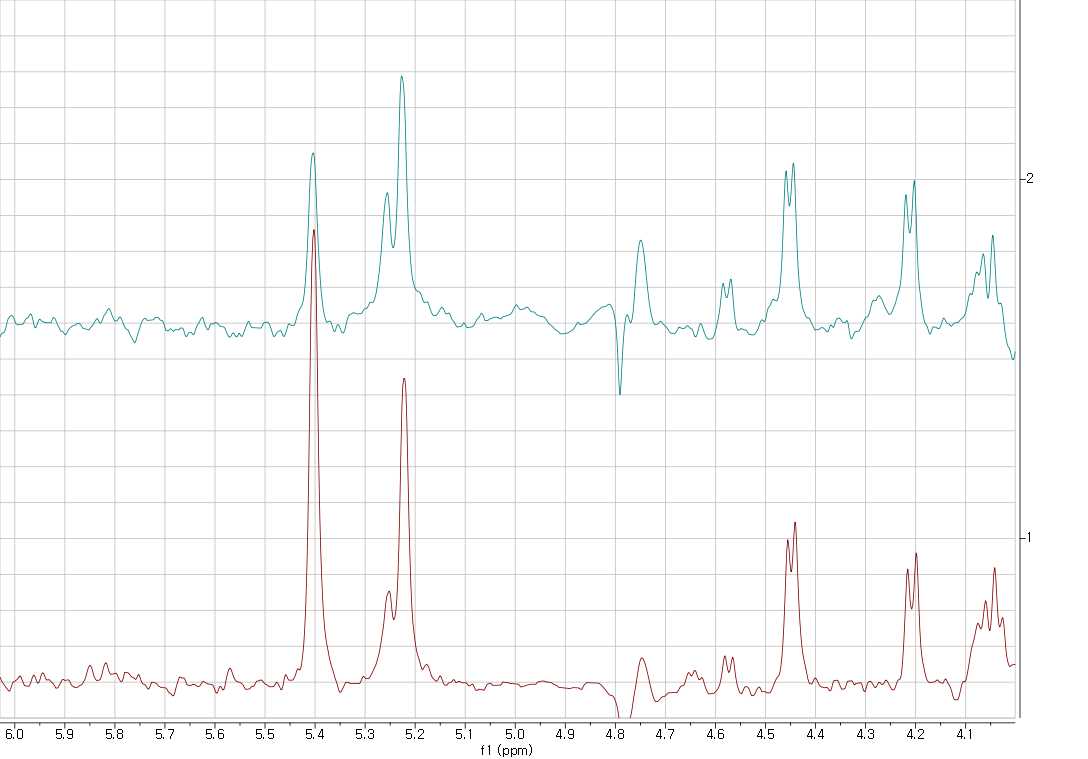


Figure S3(a).


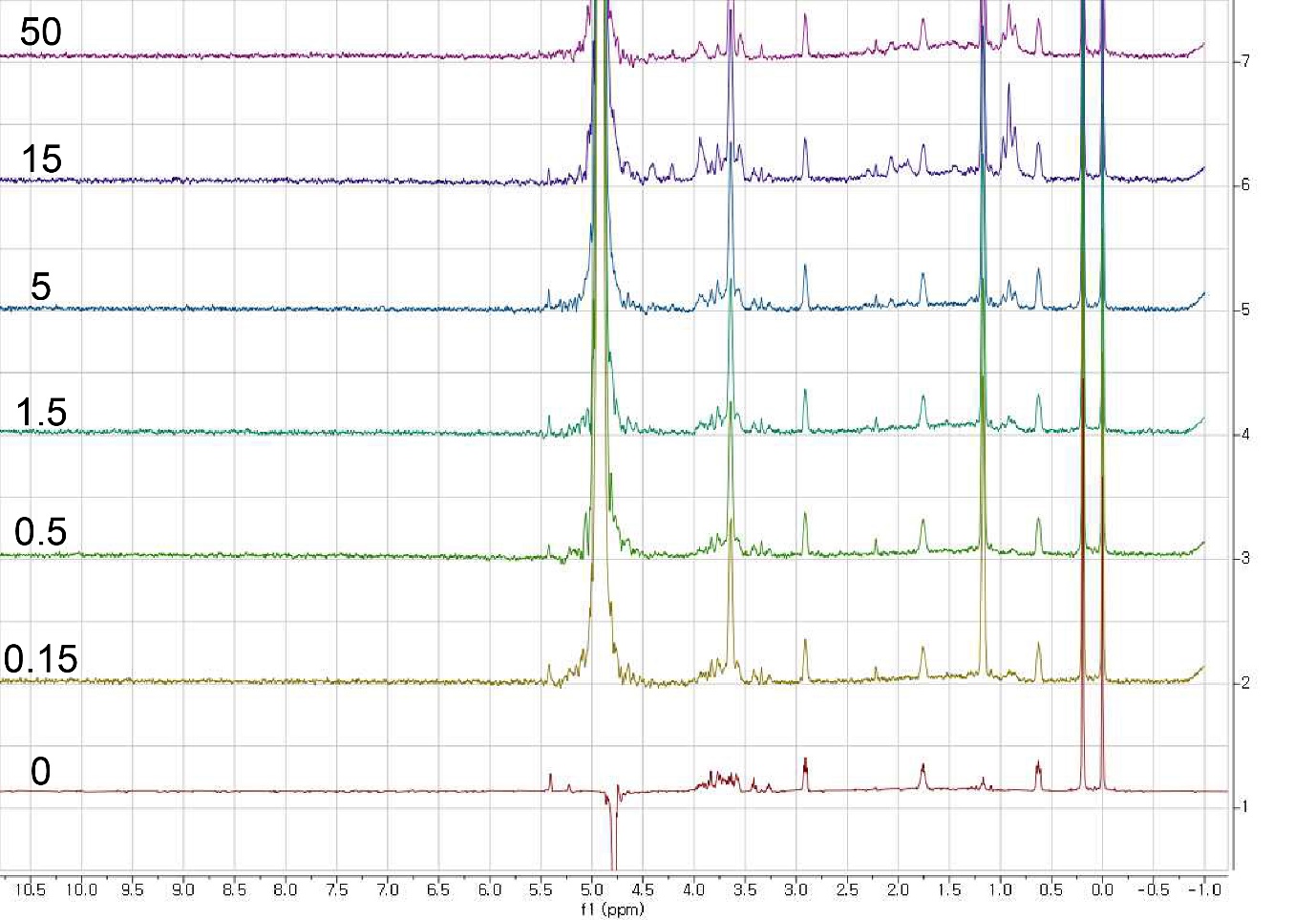


Figure S3(b).


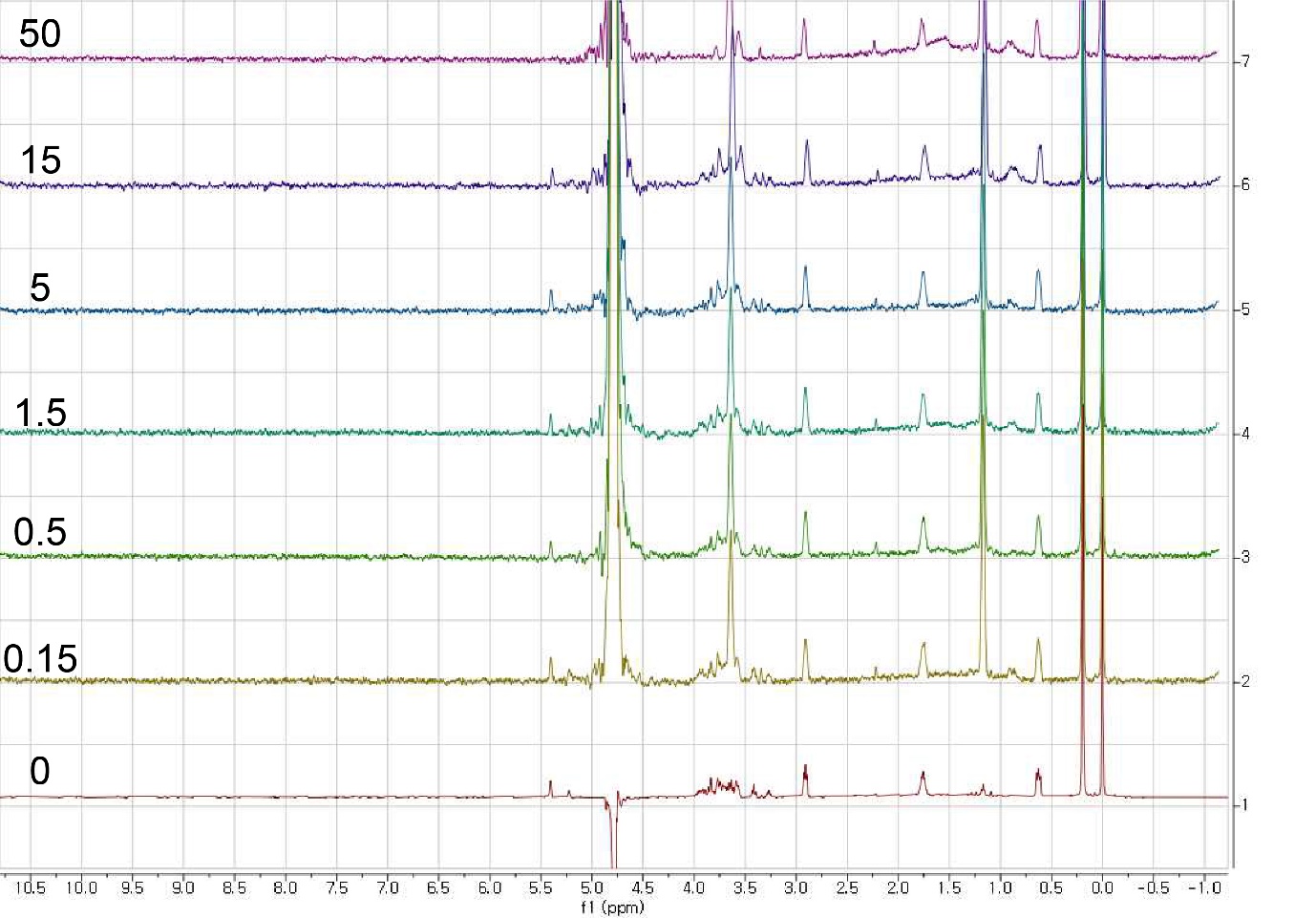


Figure S4.


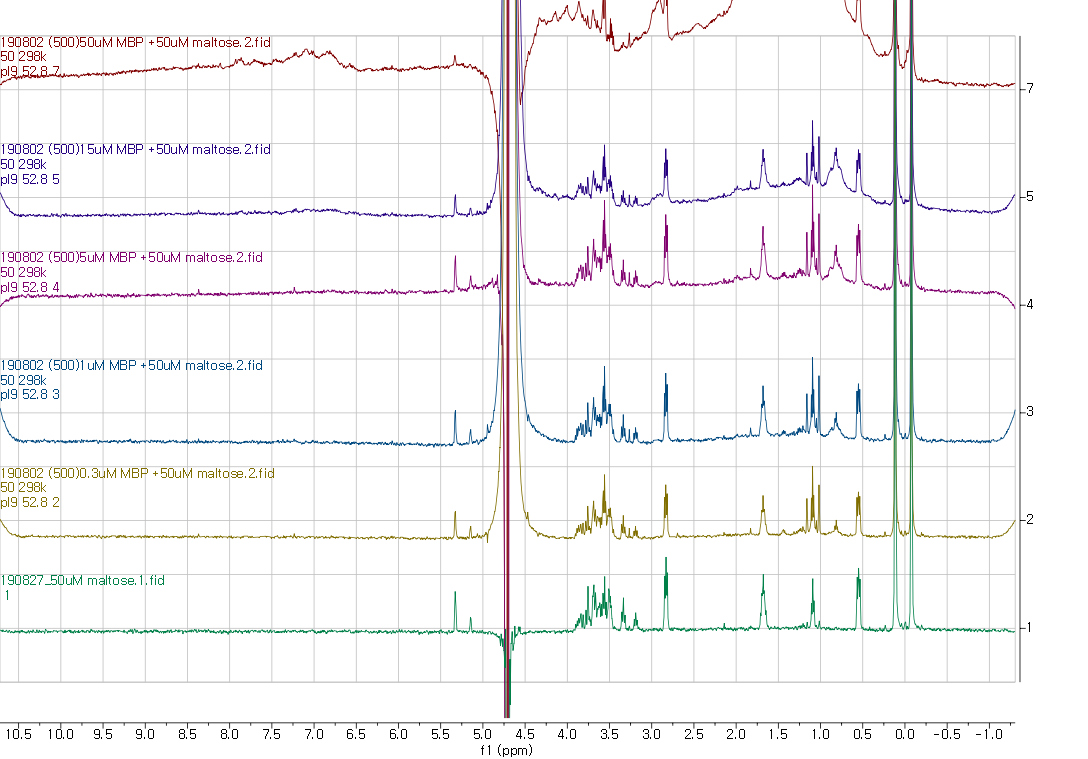


Figure S5.

Figure S6.


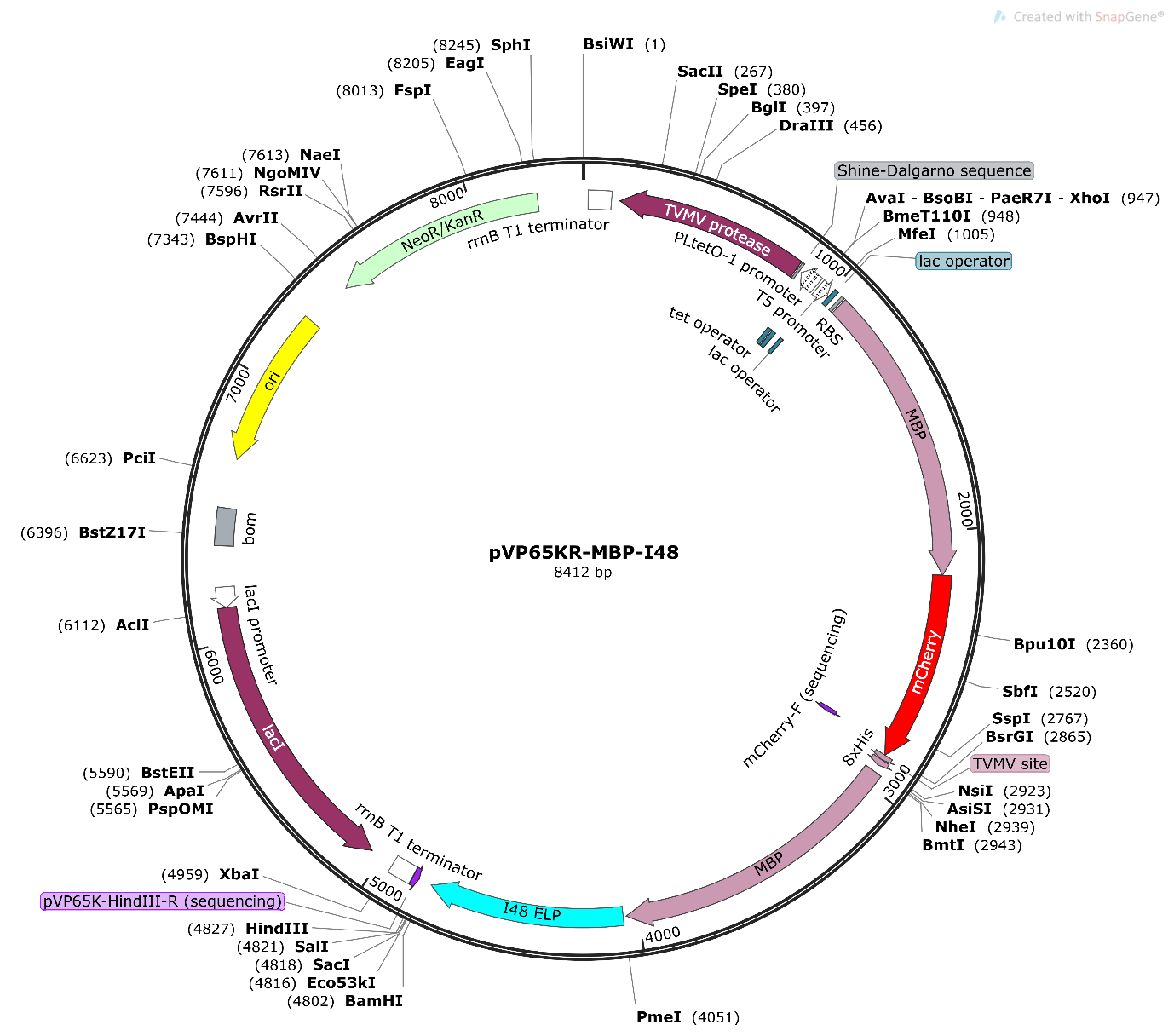

Supplement: Supplementary file 1 — Supplementary material 1 (DOCX 2102.4 kb) [file 10858_2021_381_MOESM1_ESM.docx]
